# Digital Hebrew Paleography: Script Types and Modes

**DOI:** 10.3390/jimaging8050143

**Published:** 2022-05-21

**Authors:** Ahmad Droby, Irina Rabaev, Daria Vasyutinsky Shapira, Berat Kurar Barakat, Jihad El-Sana

**Affiliations:** 1 Department of Computer Science, Ben-Gurion University of the Negev, Be’er Sheva 8410501, Israel; dariavas@post.bgu.ac.il (D.V.S.); berat@post.bgu.ac.il (B.K.B.); el-sana@cs.bgu.ac.il (J.E.-S.); 2 Department of Software Engineering, Shamoon College of Engineering, Be’er Sheva 8410802, Israel; irinar@ac.sce.ac.il

**Keywords:** digital paleography, handwritten style analysis, Hebrew medieval manuscripts, script type classification, deep-learning based classification, convolutional neural network

## Abstract

Paleography is the study of ancient and medieval handwriting. It is essential for understanding, authenticating, and dating historical texts. Across many archives and libraries, many handwritten manuscripts are yet to be classified. Human experts can process a limited number of manuscripts; therefore, there is a need for an automatic tool for script type classification. In this study, we utilize a deep-learning methodology to classify medieval Hebrew manuscripts into 14 classes based on their script style and mode. Hebrew paleography recognizes six regional styles and three graphical modes of scripts. We experiment with several input image representations and network architectures to determine the appropriate ones and explore several approaches for script classification. We obtained the highest accuracy using hierarchical classification approach. At the first level, the regional style of the script is classified. Then, the patch is passed to the corresponding model at the second level to determine the graphical mode. In addition, we explore the use of soft labels to define a value we call squareness value that indicates the squareness/cursiveness of the script. We show how the graphical mode labels can be redefined using the squareness value. This redefinition increases the classification accuracy significantly. Finally, we show that the automatic classification is on-par with a human expert paleographer.

## 1. Introduction

Historical manuscripts constitute valuable information explicitly via their textual content and implicitly through their writing material, handwriting style, and other features. Paleography is the study of ancient and medieval handwritings. The term is derived from the Greek words palaios (“old”) and graphein (“to write”). Paleography is important for understanding, authenticating, and dating historical texts.

**Figure 1:**
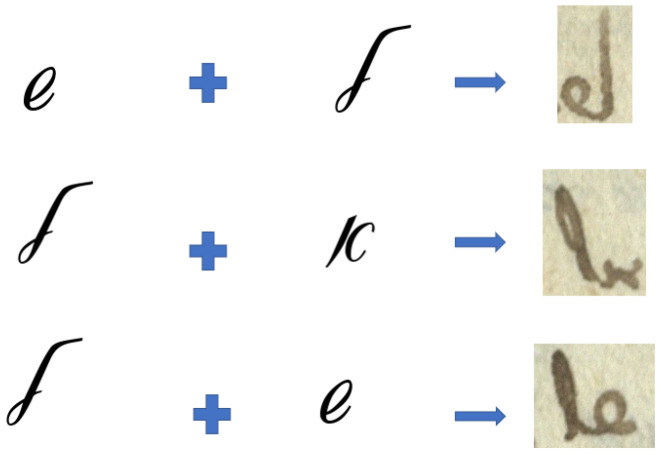
Examples of ligatures used in Hebrew ancient and medieval manuscripts that are not in use today. Two letters are joined together into a single glyph.

During the last decades, many archives and libraries’ collections were digitized. However, processing these collections requires participation of an expert due to the following: (1) reading historical texts demands knowledge of their grammar and dialectical variants, e.g., grammars of the same language from 10th, 15th, and 21st centuries are different; (2) the shape of letters changed and evolved over the time, i.e., many symbols look unfamiliar and strange to modern reader; (3) medieval manuscripts made use of many abbreviations and ligatures (letters joined together) that are not in use nowadays (see examples in Figure 1). A trained paleographer can only describe a limited number of manuscripts, and the number of such paleographers is very small. Moreover, to become a paleography expert, one needs many years of hands-on experience. Many digital collections use outdated catalogs and even uncatalogued collections. Therefore, the processing must be automated.

With advancements in image analysis and computer vision, automatic methods enhanced manual handwriting analysis. Document image processing can be divided into classical machine learning techniques that require prior features selection, and deep neural network-based techniques where features are learned inside a network itself. The most successful recent image classification approaches are deep learning-based.

One of the primary advantages of deep learning over traditional machine learning algorithms is the ability of the model to learn features directly from the data. This can save scientists weeks and months of work. Additionally, neural networks can reveal novel, more complicated features that a human expert may overlook. That is why in our research we utilize deep-learning technology for paleographic analysis of medieval Hebrew manuscripts.

The main contributions of this study include:

- A deep-learning-based classification of Hebrew manuscripts into 14 categories according to the script types and graphical modes. To train a deep neural network, we compiled a dataset of manuscripts that include these categories. To the best of our knowledge, this is the first dataset that includes samples of major Hebrew writing types and modes to address digital paleography community;
- Two different ground truth labeling schemes—hard-labeling and soft-labeling—for training a deep-learning model. The margins between the categories of writing styles are sometimes fuzzy and overlap on a visual appearances level. To categorize the document, paleographers examine the visual appearance of the handwriting as well as the codicological data, e.g., the media on which the document was written. Since we are working with digital images only, we cannot utilize codicological data. We hypothesize that hard-labeling may not be the ideal way for training a deep-learning model to recognize the writing style. Therefore, for each page image, we decided to add an additional level of labeling—a soft label. The soft label is a label vector, where each element indicates the similarity of the document’s script to a specific script type or mode. We experimentally compare and analyze hard and soft label results, and discuss the issues of paleographic analysis of Hebrew writings;
- An evaluation of the performance of deep-learning model against the human-level performance in classifying Hebrew script type. We show that deep-learning models achieve classification accuracy that is close to the expert’s.

This paper is organized as follows. We start by describing the related work. After that we provide a brief background on Hebrew paleography. Then, we move to describing methodology and experiments, starting with data collection and classification, followed by the description of the experimental setup. Then we present our finding, analyze the results, and compare them versus human-level performance. Finally, we draw conclusions and future work directions.

## 2. Related Work

Throughout the last decade, various computer vision algorithms have been applied/adapted for paleography analysis. Earlier techniques relied on hand-crafted features, which were often based on textural and grapheme-based descriptors, and their combination [1,2,3]. During the recent years, deep learning approaches have set new benchmarks in a variety of academic fields, and have also been adapted for paleographic analysis [4,5,6,7,8]. Keglevic et al. [9] proposed to use a triplet CNN to measure the similarity of two image patches. Abdalhaleem et al. [10] investigated in-writer differences in manuscripts. Their methodology is built on Siamese convolutional neural networks, which are trained to recognize little differences in a person’s writing style. Studer et al. [11] explored the effect of ImageNet pre-training for various historical document analysis tasks, including style categorization of Latin manuscripts. They experimented with VGG19 [12], Inception V3 [13], ResNet152 [14], DenseNET12 [15], and additional well-known architectures. The models trained from scratch achieved 39–46% accuracy rate, whereas the pre-trained models achieved a 49–55% accuracy rate.

Two major competitions on the categorization of medieval handwritings in Latin script [16,17] were organized in 2016 and 2017. The goal of the competitions goal was to classify medieval Latin scripts into 12 categories based on their writing styles. The findings reveal that deep learning models can accurately recognize Latin script types with more than 80% accuracy on homogeneous document collections and about 60% accuracy on heterogeneous document collections.

There have been few works on Hebrew document paleography. Wolf et al. [18] explored handwriting matching and paleographic classification, focusing on the documents from the Cairo Genizah collection. Dhali et al. [19] used textural and grapheme-based features with support vector regression to determine the date of ancient texts from the Dead Sea Scrolls collection. Ben Ezra et al. [20] trained a model for establishing the reading order of the main text by detecting insertion markers that indicate marginal additions. They used a corpus of 17 manuscripts of Tannaitic Rabbinic compositions dated from the 10th to 15th centuries. The international Israeli and French team [21,22] worked on a project that combined handwritten text recognition of Medieval Hebrew documents with a crowdsourcing-based process for training and correction the HRT model. Their project focused on a subset of rabbinic works dated to 1–500 CE.

The aforementioned projects have worked on different datasets and document types, each addressing a different aspect of the challenge. These efforts work in tandem to achieve the ultimate goal of recognizing handwritten writing in historical manuscripts. In this study, we utilize deep learning models to classify medieval Hebrew manuscripts according to their script types and modes.

## 3. Hebrew Paleography

Manuscripts are studied by means of paleography and codicology that explore the writing and the material on which manuscripts are written, respectively. The theoretical basis of Hebrew paleography and codicology are formulated in the works of Malachi Beit-Arié, Norman Golb, Benjamin Richler, and Colette Sirat [23,24,25,26,27,28,29].

Hebrew manuscripts refers to manuscripts written in Hebrew characters, as the language was often adopted from the host societies (Ladino, Judeo-Arabic, Yiddish, etc.). Geographically, the spread of the Hebrew manuscripts was larger than Latin, Greek, or Arabic manuscripts. Hebrew scripts themselves were influenced by the local traditions and often resemble the manuscripts of the host societies in scribal manner, material, and ways of production.

**Figure 2:**
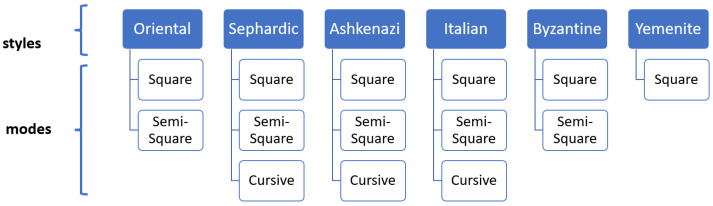
Hebrew script styles and modes; not all regional styles have cursive or semi-square mode.

**Figure 3:**
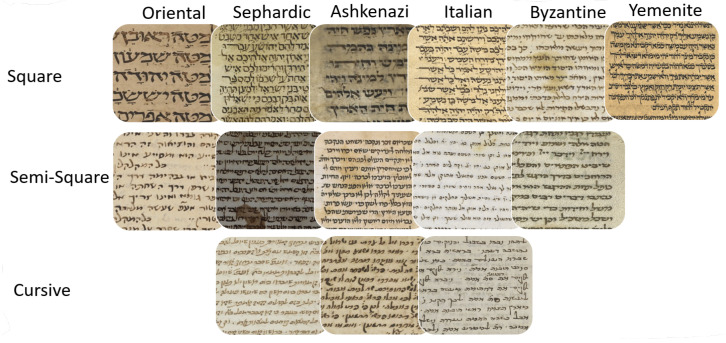
Examples of document image patches with different Hebrew script styles and modes.

There are six main types of the Hebrew script: Oriental, Sefardic, Ashkenazi, Italian, Byzantine, and Yemenite. Each script type may contain up to three graphical modes: square, semi-square, and cursive. The writing styles of Hebrew manuscripts can be classified into two branches based on their geographic origin. Oriental, Sefardic, and Yemenite styles developed in Islamic regions and were influenced by the Arabic calligraphy, while Ashkenazi and Italian styles evolved in Europe and were somewhat influenced by Latin scripts. The Byzantine type displays hybrid influences and most likely the influences of Greek scripts. Figure 2 summarizes the main Hebrew script styles and modes and Figure 3 presents sample patches for each class. Semi-square (semi-cursive) and cursive modes in each regional style are defined relatively to the style’s square mode. A graphical mode in one regional style can even look similar to a different mode in another geographical style, e.g., square in Italian can be similar to Ashkenazi semi-square. Styles and modes of Hebrew scripts, paleographically, can not be separated.

Our project aims to recognize the main types of the Hebrew script and their modes (square, semi-square, cursive). Paleographically, the backbone of our research is the SfarData (https://sfardata.nli.org.il/ (1 March 2022)), which is a project for the Hebrew paleography and codicology started in the 1970s by Malachi Beit-Arié—one of the leading Hebrew paleographers. Sfardata aimes to locate and classify all existing dated Hebrew manuscripts written before 1540. Today it includes almost 5000 manuscripts. Malachi Beit-Arié and his team met with our team, discussed our project, gave us their full support, and allowed us to use their database in its entirety. Our team’s paleographer, who is herself a student of Malachi Beit-Arié, handpicked digitized pages from the manuscripts described in the SfarData as the raw material for our project.

## 4. Vml-Hp-Ext Dataset Description

The Hebrew paleography dataset is a valuable resource both for creating a large-scale paleographic examination of Hebrew manuscripts, and assessing and benchmarking script classification methods. In this paper we present an extended VML-HP-ext (Visual Media Lab—Hebrew Paleography-Extended) dataset. Compared to the first version presented in [30], the extended dataset includes sample pages from three times more manuscripts. Every manuscript was carefully selected by our team’s paleographer. The majority of the manuscripts used in this dataset are kept in the National Library of Israel, the British Library, and the Bibliothéque nationale de France. Almost all manuscripts in the Oriental square script belong to the National library of Russia (we used b/w microfilms from the collection of the Institute for Microfilmed Hebrew Manuscripts at the National Library of Israel). To simplify ground truth labeling, we only included pages with one script type and one script mode per page. For example, Sephardic square only, and not main text in Sephardic square and comments in Sephardic cursive. The main challenge when compiling the dataset was the limited amount of available digitized manuscripts. For some script types (Italian, Byzantine) the shortage was more pronounced; for others (Ashkenazi, Sephardic) we had manuscripts in abundance. Keeping the dataset balanced was a challenge in itself.

The enlarged VML-HP-ext collection contains 715 page images excerpted from 171 different manuscripts. We also provide the official split of the VML-HP-ext into training, typical test, and blind test sets. A typical test set includes unseen pages of the manuscripts from the training set. While the training and typical test sets are disjoint on the page level, they do share the same set of manuscripts. Therefore, we also provide the blind test set, which consist of manuscripts that do not appear in the training set. The blind test set imitates a real-life scenario, where a scholar would like to obtain a classification for a previously unseen document. Table 1 and Table 2 summarize the extended VML-HP-ext dataset.

## 5. Methodology and Experimental Setting

We view the problem of script type identification as an image classification problem, where a class is assigned to a given image. The use of deep-learning models is widely regarded as the best approach to tackle image classification. Therefore, we will use deep-learning, specifically, the Convolutional Neural Network (CNN) model to classify text images into script types.

In this section, we describe the experimental setting (model structure, data, and training scheme) used in our experiments. Having a common setting across all the experiments makes it more comparable.

### 5.1. Model

We adopt a CNN model to classify patches extracted from the pages in the dataset. The models consist of two components, an established CNN backbone (e.g., ResNet, VGG) which acts as a feature extractor, followed by two fully connected layers. We rely on established CNN architectures rather than a more complex state-of-the-art models, such as transformers [31], or build our own architecture because script type classification is relatively simple and does not require more complex models to solve it. This point is backed by the results in the experiments in Section 6.2. The CNN backbone of the models were initialized from a pretrained model on ImageNet [32].

### 5.2. Data

The models are trained and tested on patches extracted from pages in the VML-HP-ext dataset. The patches were extracted using the patch generation method proposed in our previous work [30], which extracts patches with a uniform text scale and on average 5 lines in each patch. According to our Hebrew paleography expert, in general such patches contain the necessary information for classification. The pixel values of the patches were normalized. The models were trained using 50,000 patches and tested on 13,500 patches. It is worth noting that the test patches are extracted from the blind test set, i.e., from manuscripts that do not appear in the training set. This prevents trivial solutions where the model remembers the background pattern of the pages.

### 5.3. Training

**Figure 4:**
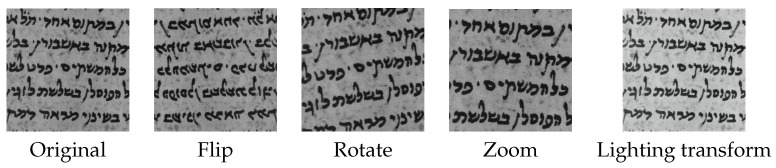
Example of augmented patches.

The training was done in two stages, (1) The CNN backbone of the model is frozen, and the model is trained for 20 epochs. (2) The CNN backbone is then unfrozen, and the whole model is trained for additional 20 epochs. We use binary cross entropy as loss function. During training, random augmentations are applied on the patches. These augmentations include flip, rotate, zoom, warp, and lighting transforms (see Figure 4).

## 6. Case Study

As mentioned in Section 4, the VML-HP-ext dataset expanded on its previous version introduced in [30]. Therefore, in the following section we first revisit experiments made on the original dataset, mainly to find the optimal input image representation and network architecture. Then, we conduct experiments to visualize relevant script features, test two approaches that split the classification of the regional style and graphical mode, and discuss new ways to classify Hebrew script types.

### 6.1. Effect of Input Image Representation

Our first experiment is to find the most appropriate input image representation. To this end, we trained a ResNet50 [14] model to classify patches of different image input representations extracted from pages in the VML-HP-ext dataset. The model is trained and tested in the same experiment setting mentioned in Section 5.

**Figure 5:**
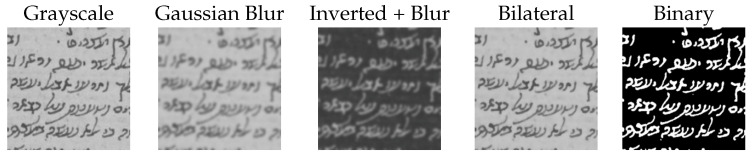
Example of the input patch types we experimented with.

We experimented using several types of input such as, raw grayscale, binary, and smoothed patches (see Figure 5). Smoothing patches by applying Gaussian and bilateral filters reduces the amount of background information, which may lead the model to overfit, i.e., it remembers the background pattern.

Table 3 shows the accuracy of the models trained using the different types of input patches. We can see that the model trained with binary patches achieves the highest accuracy of 62%, while the model trained using the raw grayscale patches achieves the lowest accuracy of 52%. This can be attributed to the fact that the model trained on the grayscale patches is learning to leverage the background pattern during classification. This point is strengthened by considering the amount of background information present in each input type. The raw grayscale patches contain the most background information, followed by patches with Bilateral and Gaussian filters, and binary patches, which eliminate the background altogether. This exact order reflects the classification accuracy. Therefore, we can conclude that the appropriate input type is binary.

### 6.2. Effect of Network Architecture

We experimented with different CNN backbone architectures of the classification model. The models are trained and tested using binarized patches. Similar to the previous experiment, the models are trained and tested under the setting mentioned in Section 5.

**Figure 6:**
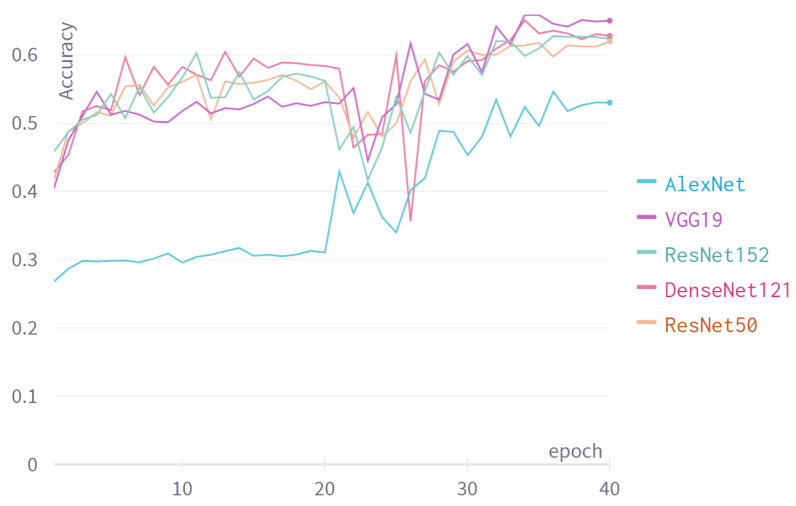
The accuracy of different models during training.

Figure 6 show the accuracy of the models on the test set during training. We can see that by the end of training, VGG19 has the best performance, reaching an accuracy of 66%. DenseNet and both ResNet versions reached similar accuracy, about 62%. AlexNet did not perform as well as the rest, reaching an accuracy of 53%. We can explain this result by considering the complexity of each model. A simple model may not have the capacity to learn the classification problem but is less likely to overfit on the training set. In contrast, a more complex model is able to learn more complex problems, but is more likely to overfit. We can conclude that VGG19 is complex enough to reach 66% accuracy and is simple enough to avoid overfitting.

### 6.3. Page Level Prediction

We used the trained patch classification models from the previous experiment to make classification at the page level. The classification is done by sampling a number of patches from the page, which are classified using the trained models. The page is classified as the majority class of the sampled patches. We experimented with the number of sampled patches to find the optimal number.

**Figure 7:**
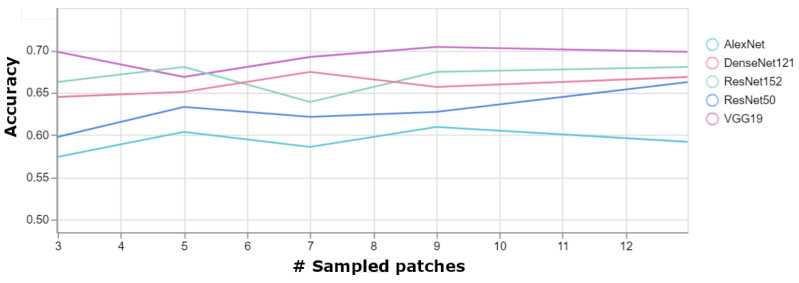
Page level accuracy as a function of the number of sampled patches per page.

Figure 7 shows the page level accuracy as a function of the number of sampled patches. We can see that the page level accuracy on average is higher by about 5% than the patch level accuracy. Unsurprisingly, VGG19 reaches the highest accuracy of 70.4%. From the figure, we can conclude that sampling 9 patches per page is enough to achieve the best results. We can see that adding more patches, beyond 9, does not improve the results further.

### 6.4. Evaluating the Best Performing Model

**Figure 8:**
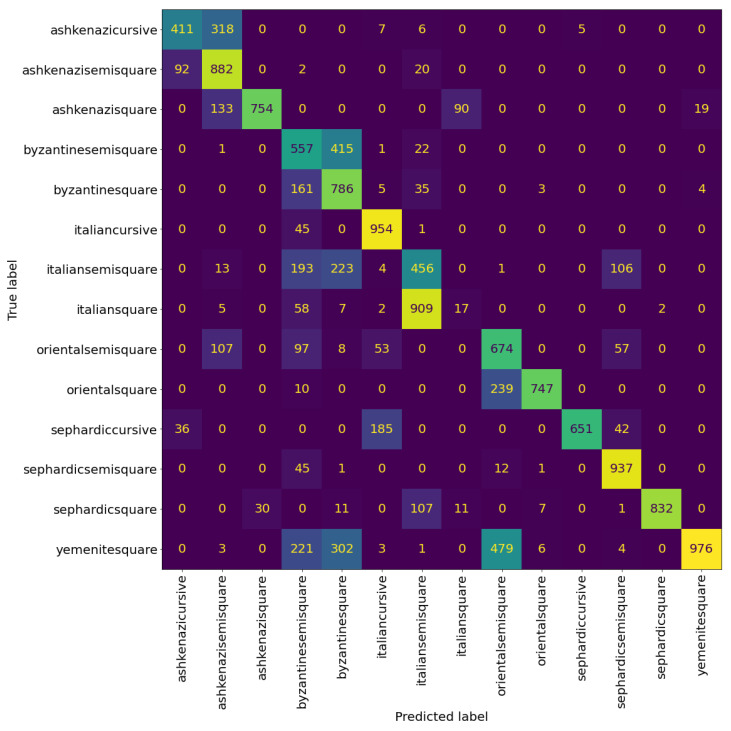
Confusion matrix of VGG19 at the patch level. The cells colour correspond to the number of patches, brighter colours indicates more patches.

**Figure 9:**
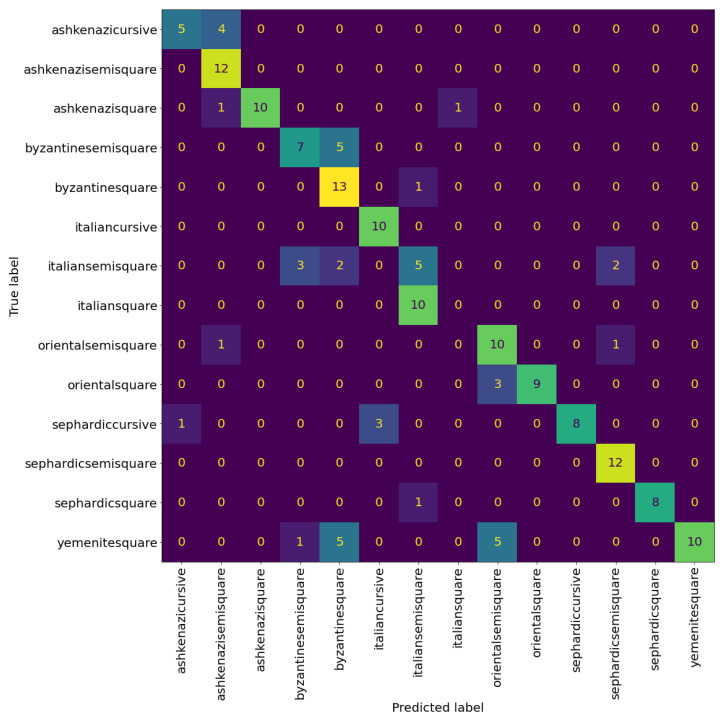
Confusion matrix of VGG19 at page level with nine sampled patches per page. The cells colour correspond to the number of patches, brighter colours indicates more patches.

**Figure 10:**
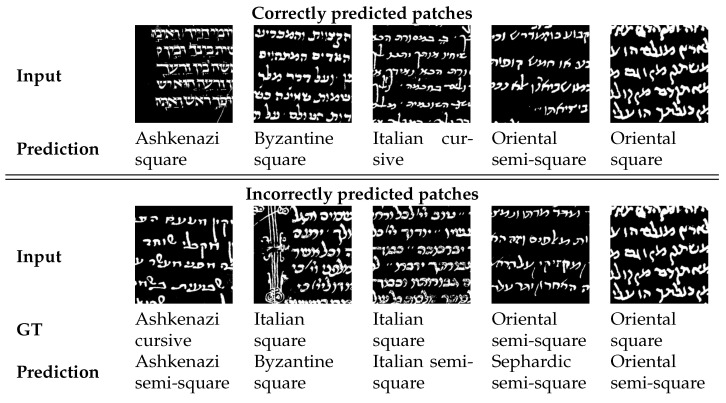
Example of classification results of the VGG19 model.

Table 4 and Table 5 show the precision, recall, and F1-score of the VGG model at the patch and page level, respectively. We can see that the model can classify the Sephardic script types very good, almost classifying all Sephardic pages perfectly. On the other hand, the model struggles to classify Italian square and semi-square. As can be seen in Figure 8 and Figure 9, a large portion of Italian semi-square patches and pages are classified wrongly as Sephardic square, Byzantine square, and Byzantine semi-square. Almost all Italian square patches/pages are classified as Italian semi-square. From both confusion matrices, we can see that apart from Sephardic, confusing between square and semi-square is common in all other regional styles. This confusion can be attributed to the fact that there is no clear-cut between square and semi-square modes, as there are script types defined as square which visually look closer to semi-square (e.g., see the Italian and Oriental square patches in Figure 10). The reason for this is that when classifying the manuscripts in SfarData, the researchers did not rely solely on paleographic criteria; they also considered codicological features such as type and kind of the material (there are different types of both parchment and paper), drawing techniques, etc. These codicological features of a manuscript are not available to the model.

### 6.5. Features Visualization

To visualize the features that guide the model toward its finial prediction, we use the Gradient-weighted Class Activation Mapping (Grad-CAM) technique [33]. Grad-Cam uses the gradients of a target class flowing into the final convolutional layers of the CNN, which in our case is VGG19, to produce a coarse localization map that highlights important regions for predicting the target class.

**Figure 11:**
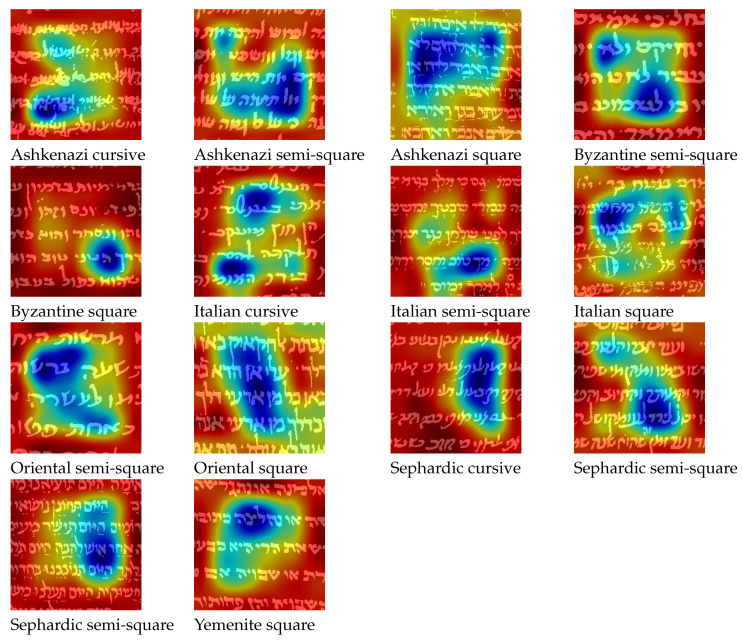
Visualization of important features for classification using Grad-CAM.

Figure 11 illustrates the important features of a sample patch for each one of the script types. We can see that in some cases the network ”looks” at specific letters in the patch, e.g., letters ‘lamed’ in Byzantine semi-square, ‘hey’ in Byzantine square, ‘bet’, ‘resh’ in Oriental semi-square, and ‘aleph’ in Oriental square. While in other cases, the network looks at the global layout of the patch, such as in all Ashkenazi types, Italian square, and to some degree Sephardic semi-square.

### 6.6. Splitting Regional Styles and Graphical Modes Classification

In the previous experiments, we saw that one of the main classification errors was due to the confusion between the different graphical modes within each regional styles. This is very prominent for Italian square, where almost all the Italian square patches are classified as Italian semi-square. In an attempt to solve this confusion, we experimented with classifying the regional styles and graphical modes separately. We trained two VGG19 models to classify the regional styles and graphical modes, respectively. The two models were trained using the same training scheme as the previous experiments (i.e., using binarized patches, and the same setting mentioned in Section 5).

**Figure 12:**
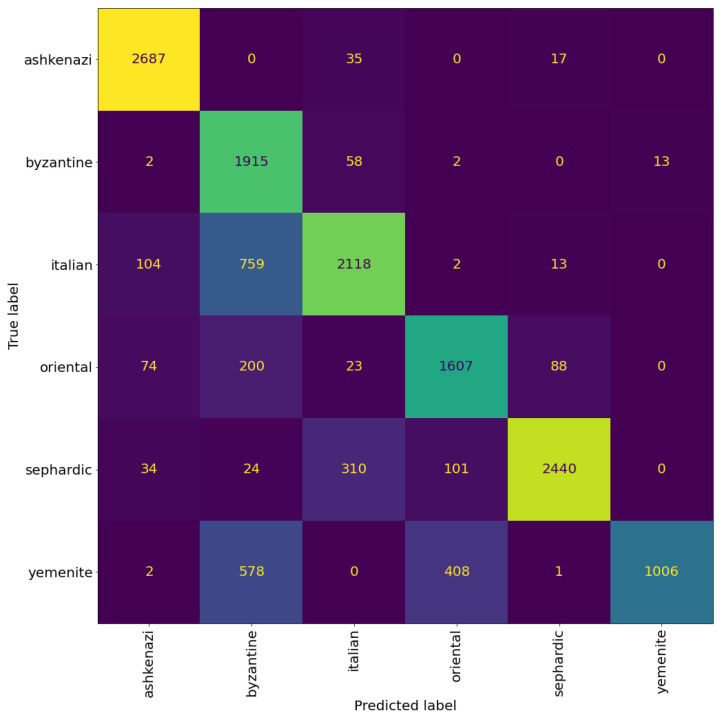
Confusion matrix of VGG19 model trained for regional style classification. The cells colour correspond to the number of patches, brighter colours indicates more patches.

**Figure 13:**
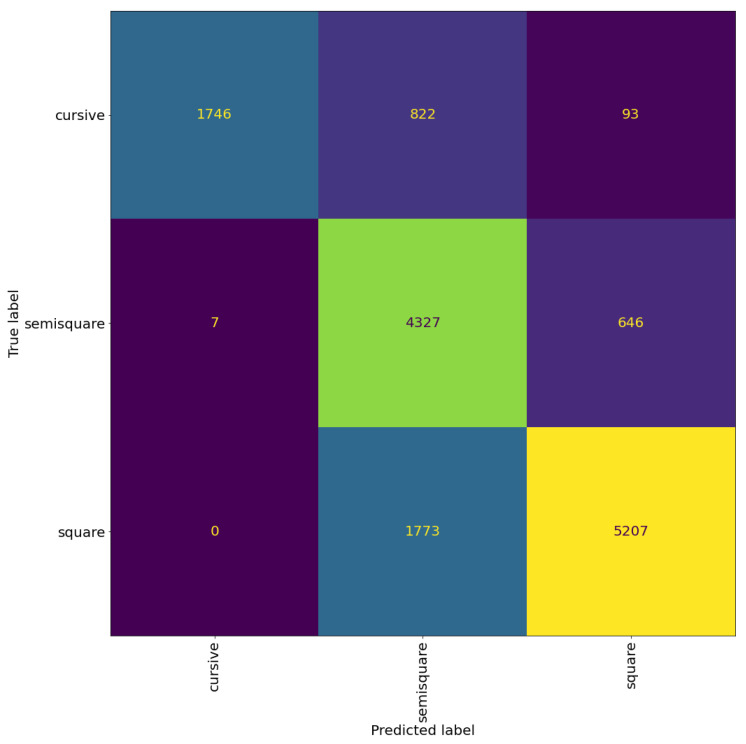
Confusion matrix of the VGG19 model for graphical mode classification. The cells colour correspond to the number of patches, brighter colours indicates more patches.

Figure 12 and Figure 13 shows the confusion matrices of the trained models. We can see that the model trained to classify the regional styles does a good job, reaching an accuracy of 81% at patch level and 85% at page level. The model trained to classify the graphical modes reached an accuracy of 77% at patch level and 80% at page level. However, we can see that there is still significant confusion between the graphical modes, suggesting that this confusion is not a limitation of the model, but rather a problem with the distinction between the graphical modes, specifically, the distinction between square and semi-square in certain regional styles (as mentioned before, see the Italian and Oriental square patches in Figure 10).

To evaluate the two models against the models trained using the original labels (regional and graphical combined), we combined the classification from the two models to get a classification result similar to the original labels. For example, suppose a patch got the prediction of Italian from the regional model and square from the graphical model, then the combined label will be Italian square. We found that in rare cases, this approach introduces labels that are not present in the dataset, e.g., Byzantine cursive, Oriental cursive, and Yemenite semi-square. This classification scheme achieved 64% accuracy at patch level and 69% accuracy at page level, indicating that classifying the regional styles and graphical modes separately only worsened the results rather than improving it.

### 6.7. Hierarchical Classification

**Figure 14:**
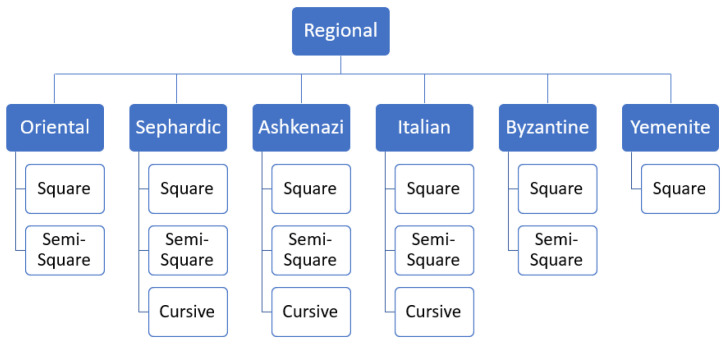
Illustration of the hierarchical classification approach. For a given patch, the regional style is first classified, then based on this classification the patch is based to the relevant model in the second layer to classify the graphical mode.

In the following experiment, we explore a hierarchical approach for classification. In this approach, patches are classified in a hierarchical manner, as the regional style is predicted at the first level, then the graphical mode is predicted by a dedicated model for each regional style. For example, if a patch was classified as Ashkenazi, then it is passed to a model trained to classify graphical modes on Ashkenazi patches (see Figure 14).

All the models in the hierarchy have VGG19 architecture, similar to the previous experiment. The models are trained using the relevant subset from the patches mentioned in Section 5, e.g., the Ashkenazi classification model at the second level was trained using patches with the Ashkenazi regional style and labeled with their graphical modes. The models are trained under the setting mentioned in Section 5.

Table 6 shows the patch level accuracy of the models across the hierarchy, the model predicting the regional styles and the models predicting the graphical modes for the regional styles (there is no need for a Yemenite model because it only has one graphical mode). We can see that while all the models achieve accuracy higher than 80%, the classification model for Italian script got 66% accuracy, with the major source of error from classifying square as semi-square.

**Figure 15:**
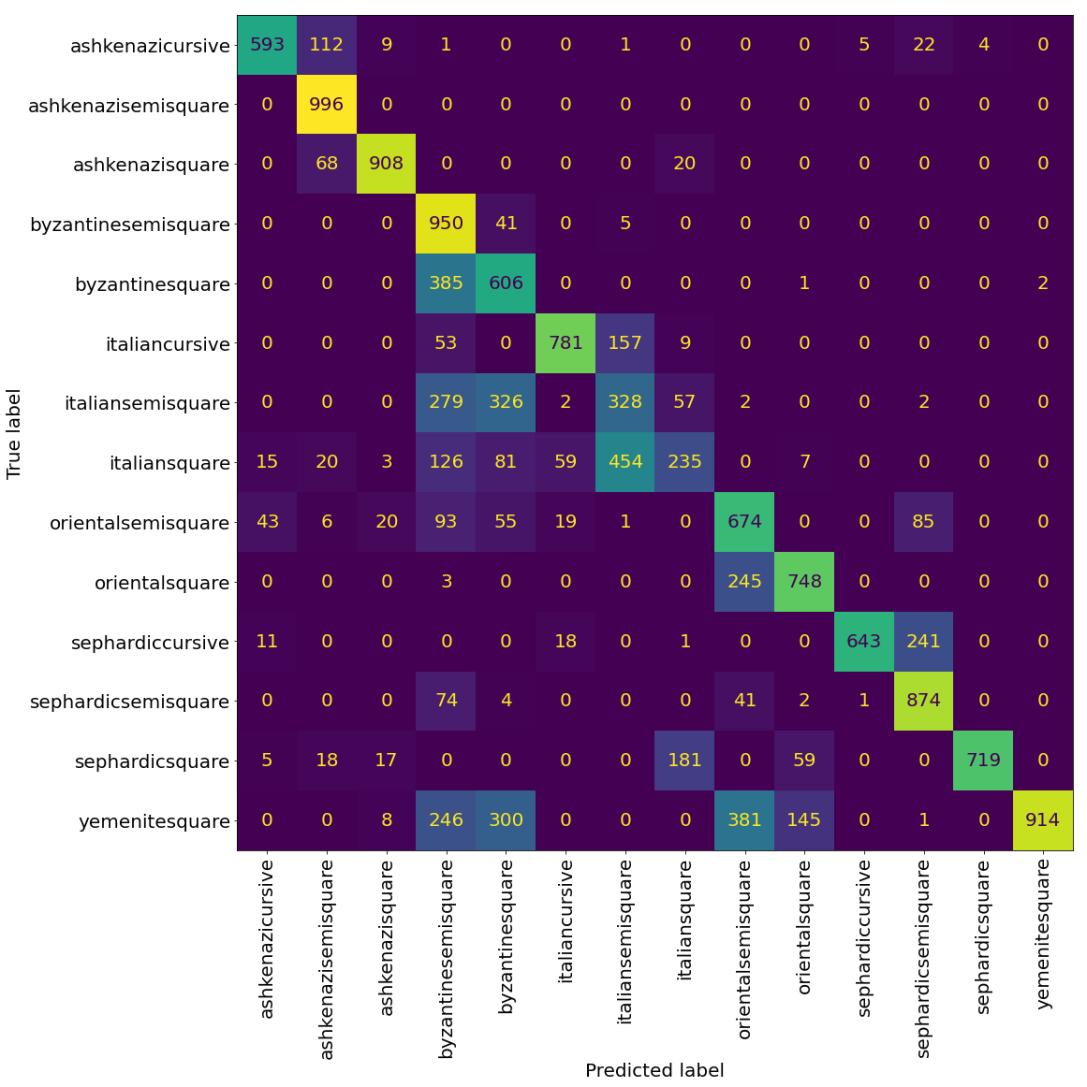
Confusion matrix of the Hierarchical model at patch level. The cells colour correspond to the number of patches, brighter colours indicates more patches.

Table 7 shows the evaluation results of the hierarchical model at patch level. There is noticeable improvement over the previous experiments on almost every metric. The model achieves an accuracy of 68%, two percentage points over the accuracy of the best performing model in the previous experiments. A significant source of this improvement came from increasing the classification accuracy of the Italian square. However, it is still a challenge, as can be seen in Figure 15 compared to Figure 8.

**Figure 16:**
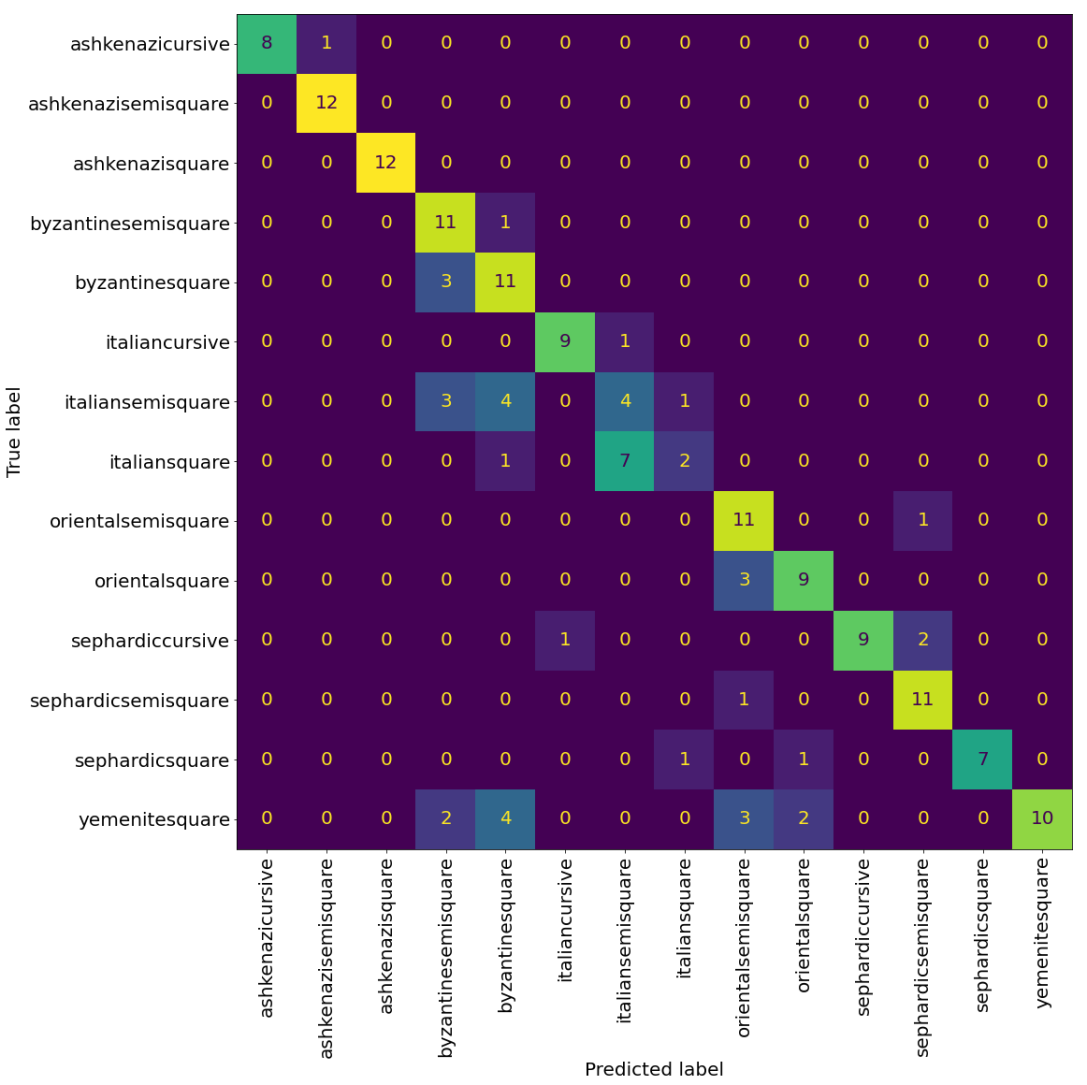
Confusion matrix of the hierarchical model at page level with nine patches sampled per page. The cells colour correspond to the number of patches, brighter colours indicates more patches.

Table 8 reports the evaluation results of the hierarchical model at page level. The hierarchical model achieves the best evaluation results on page level as well, getting an accuracy of 75%. From Figure 16 we can see that the hierarchical model reduces the confusion between the graphical modes, more notably in Byzantine and Italian scripts.

### 6.8. Soft-Labeling: Reconsidering Graphical Mode Classes

We saw from the previous experiments that the distinction between the graphical modes (i.e., square, semi-square, and cursive) is not always well-defined. We hypothesize that the graphical mode of the script is not a discrete class, but rather a spectrum where “Squareness” and “Cursiveness” are at the two ends, and the manuscripts carry features from multiple scripts. Therefore, we added a soft-labeling scheme to the dataset, where each manuscript is labeled using a vector of size eight. The first six elements of the vector express the degree of similarity of the manuscript to belong to a certain regional type (Ashkenazi, Italian, Sephardic, Oriental, Byzantine, and Yemenite) and the last two elements are the degrees of similarity to a certain graphical mode, square, and cursive (similar values for both square and cursive indicate the semi-square mode). Here, we focus only on the soft-labeling of the graphical modes.

**Figure 17:**
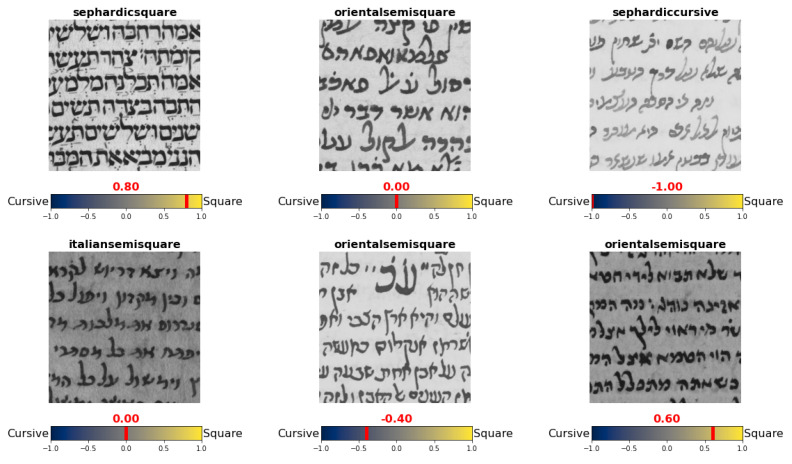
Sample patches with their respective graphical mode soft-labels. Above each patch the original script type label appears, and below it the graphical mode spectrum with soft-label indicated in red appears.

In this experiment, we converted the soft-labeling of the graphical modes into a value on the spectrum [−1,1], where a value of −1 indicates that the script is cursive, and 1 indicates that it is square (as illustrated in Figure 17). We refer to this value as *squareness value*. Using the obtained squareness values, we trained a regression model with VGG19 backbone. The model was trained and tested similar to the previous experiments (i.e., using binary patches, and the same setting mentioned in Section 5), the only difference is that the patches are labeled with their squareness values.

**Figure 18:**
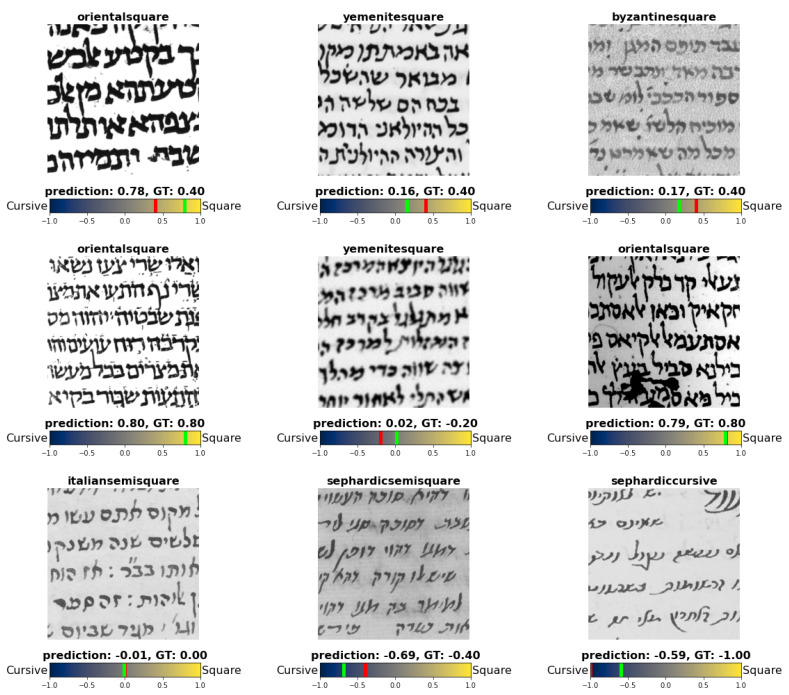
Sample prediction of the graphical mode soft-labeling using the regression model. Above each patch its original label appears. Below each patch appears the prediction of the model indicated in green, and the ground-true (GT) soft-label indicated in red. In cases where the red line does not appear, it means that the two line are on top of each other.

To evaluate the model numerically, we calculated the Root Mean Square Error (RMSE) on the test set. RMSE is calculated according to Equation (1), where y(i) is the predicted label for patch *i*, and $\hat{y}\left(i\right)$ is its actual label. The model achieved RMSE of about 0.32, meaning that the prediction of the model is on average 0.32 away from the true label. We can see the quality of the predictions in Figure 18.

(1) $$\begin{array}{c} RMSE=\sqrt{\frac{\sum_{i=0}^{N}||y\left(i\right)-\hat{y}\left(i\right){\left|\right|}^{2}}{N}} \end{array}$$

The squareness value allows us to redefine the graphical in a more precise manner. To test this, we redefined the graphical mode of each script type, based on its squareness value, as follows:

$$\begin{array}{c} Graphical\left(s\right)=\left\{\begin{array}{cc} \mathrm{semi}\text{-}\mathrm{square}, & Squareness(s)\in [-t,t]\\ \mathrm{square}, & Squareness(s)>t\\ \mathrm{cursive}, & Squareness(s)<-t \end{array}\right. \end{array}$$

**Figure 19:**
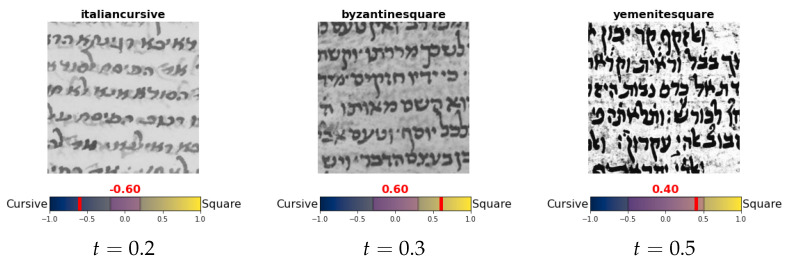
Illustration of how semi-square can be redefined using different values of *t*. The purple region in the middle indicates the squareness value range that may be considered semi-square. A text with squareness value in the purple region will be considered semi-square, squareness value to the right of this region is considered square, and to the left is considered cursive.

In words, given a value *t* (semi-square region length) between zero and one, a script type with squareness value between [−t,t] is defined as semi-square, squareness value grater than *t* is defined as square, and less than −t is defined as cursive (as illustrated in Figure 19).

**Figure 20:**
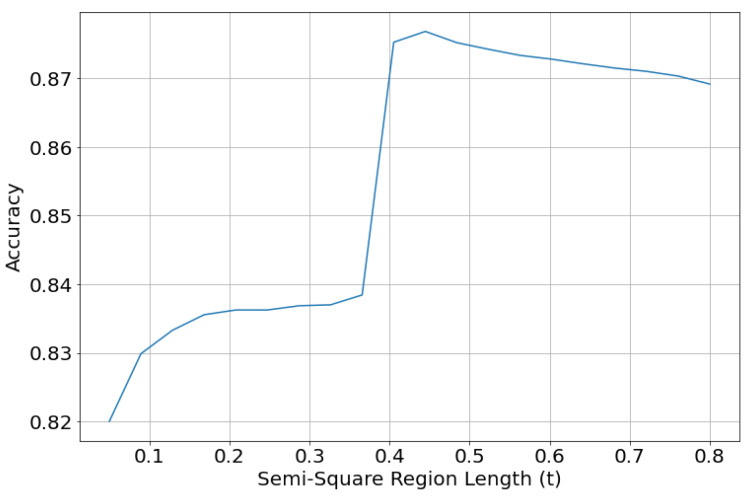
Accuracy of the combined results of the trained regression model and regional style classifier. The accuracy is measured with different values of *t*.

Redefining the graphical modes using the squareness value affects the evaluation of our trained models. We combine the predictions of the trained regression model and the regional style model from the previous experiment, to obtain both regional style and graphical mode. The obtained results were evaluated on the test set with the redefined graphical modes using different values of *t* (semi-square region length). Figure 20 shows the accuracy of the results obtained using the regression model and the regional classifier from the previous experiment. We can see that redefining the graphical modes drastically increases the accuracy compared with the models in our previous experiments; going from 66% accuracy of the best performing VGG19 model to greater than 82% accuracy. The accuracy of the results ranges from 82% for t=0.05 to around 87% for t>0.4.

### 6.9. Comparing Deep Neural Network Performance against a Paleographer Expert

In the previous sections, we reported performances of deep learning model in several settings. However, to evaluate the effectiveness of the model, we need to compare its accuracy versus the accuracy of an expert. For this, we compiled an online questionnaire (https://forms.gle/5QgGQ6x53tt7tkmn6 (1 March 2022)), and asked a paleographer to classify 75 document patches according to the classes of script types and modes. The document patches were randomly chosen from the set of patches used in our experiments, approximately five from each class. The paleographer who participated in this experiment is the coordinator of the manuscripts’ reading room at the National Library of Israel—one of today’s leading and most experienced Hebrew paleographers. The accuracy rate of the paleographer expert is 70%. We can draw two conclusions from this experiment. First, the problem was challenging for the human expert due to the unusual format: paleographers work with manuscripts and pages, not patches. Second, the automatic classification achieves human-level performance. A potential limitation of this experiment is the number of participants, which was one paleographer. Unfortunately, the number of Hebrew paleographers is extremely small, and exempt the paleographers who created the SfarData. However, we do not expect a larger experiment to change the results significantly.

## 7. Conclusions

In this paper, we explored automatic scripts styles and modes classification using deep-learning techniques. For this, we compiled the VML-HP-ext dataset to train and test our approaches. The VML-HP-ext dataset contains carefully curated pages extracted from manuscripts of SfarData collection. The experiments were conducted by analyzing and classifying patches extracted from the manuscripts pages. We used a CNN-based deep learning models to classify and analyze the patches.

We conducted experiments to find the appropriate input image representation and network architecture for script type classification. Binary patches provide the best results, because binarization eliminates background information, which reduces the likelihood that the model overfits on the training data.

According to our experiments, the VGG19 outperforms other CNN networks, achieving an accuracy of 66% at patch level. In addition, we tested the classifying pages, where a number of patches are sampled at random from a page and the page is classified as the majority class of the sampled patches. We found out that the page accuracy is higher by about 5% than the patch level accuracy, where VGG19 reached an accuracy of 70% at a page level with 9 patches sampled per page. Furthermore, we experimented with classifying the regional styles (Ashkenazi, Byzantine, Italian, etc.) and graphical modes (square, semi-square, and cursive) separately. First, we used two independent models—one that classifies the regional styles and another that classifies the graphical modes. Then, we tried a hierarchical classification approach, which includes two levels. The first level consist of a model that classifies the regional styles, and the second level contains six models that classify the graphical modes. The hierarchical approach achieved the highest accuracy of 68% at patch level and 75% at page level.

We also explored using soft-labels to define a value we call squareness value, which indicates the squareness/cursiveness of the script. Using the squareness value, we showed how the graphical mode labels can be redefined, and this redefinition increases the classification accuracy from 68% to 82–87%. Finally, we compared the automatic classification versus human expert, and showed that automatic classification is on-par to human-level performance.

## Figures and Tables

**Table 1:** Summary of the extended VML-HP-ext dataset. Some scripts do not have semi-cursive or cursive modes. Mss = manuscripts, pp = pages.

Type	Mode					
	Square		Semi-Square		Cursive	
	#Mss	#pp	#Mss	#pp	#Mss	#pp
Ashkenazi	14	56	12	48	12	48
Byzantine	7	49	12	48	-	-
Italian	5	50	11	44	5	50
Oriental	15	45	11	44	-	-
Sephardic	15	45	16	48	12	48
Yemenite	24	92	-	-	-	-

Cells with the minus sign (“-”) indicates that the corresponding script type does not exist.

**Table 2:** The VML-HP-ext dataset—official split. Mss = manuscripts, pp = pages.

	Set	# Mss	# pp
	train	130	400
	typical test	130	143
	blind test	41	172
	total	171	715

**Table 3:** The accuracy of the model trained using different patch types.

	Input Type	Accuracy
	Grayscale	52%
	Gaussian Blur	57%
	Inverted + Gaussian Blur	57.5%
	Bilateral	54%
	Binary	62%

**Table 4:** Evaluation results of the VGG19 model at patch level.

Label			Precision		Recall		F1-Score
Ashkenazi cursive			0.76		0.55		0.64
Ashkenazi semi-square			0.60		0.88		0.72
Ashkenazi square			0.60		0.88		0.72
Byzantine semi-square			0.40		0.56		0.47
Byzantine square			0.45		0.79		0.57
Italian cursive			0.79		0.95		0.86
Italian semi-square			0.29		0.46		0.36
Italian square			0.14		0.02		0.03
Oriental semi-square			0.48		0.68		0.56
Oriental square			0.98		0.75		0.85
Sephardic cursive			0.99		0.71		0.83
Sephardic semi-square			0.82		0.94		0.87
Sephardic square			1.00		0.83		0.91
Yemenite square			0.98		0.49		0.65
**Accuracy**							0.66
**Macro avg**			0.69		0.67		0.65
**Weighted avg**			0.71		0.66		0.65

**Table 5:** Evaluation results of the VGG19 model at page level with nine patches sampled per page.

Label			Precision		Recall		F1-Score
Ashkenazi cursive			0.83		0.56		0.67
Ashkenazi semi-square			0.67		1.00		0.80
Ashkenazi square			1.00		0.83		0.91
Byzantine semi-square			0.64		0.58		0.61
Byzantine square			0.52		0.93		0.67
Italian cursive			0.77		1.00		0.87
Italian semi-square			0.29		0.42		0.34
Italian square			0.00		0.00		0.00
Oriental semi-square			0.56		0.83		0.67
Oriental square			1.00		0.75		0.86
Sephardic cursive			1.00		0.67		0.80
Sephardic semi-square			0.80		1.00		0.89
Sephardic square			1.00		0.89		0.94
Yemenite square			1.00		0.48		0.65
**Accuracy**							0.70
**Macro avg**			0.72		0.71		0.69
**Weighted avg**			0.73		0.70		0.69

**Table 6:** The patch level accuracy of the models in the hierarchy.

Model		Accuracy
	Regional	81%
Graphical modes		
	Ashkenazi	93%
	Byzantine	79%
	Italian	66%
	Oriental	88%
	Sephardic	91%

**Table 7:** Evaluation results of the hierarchical model at patch level.

Label			Precision		Recall		F1-Score
Ashkenazi cursive			0.89		0.79		0.84
Ashkenazi semi-square			0.82		1.00		0.90
Ashkenazi square			0.94		0.91		0.93
Byzantine semi-square			0.43		0.95		0.59
Byzantine square			0.43		0.95		0.59
Italian cursive			0.89		0.78		0.83
Italian semi-square			0.35		0.33		0.34
Italian square			0.47		0.23		0.31
Oriental semi-square			0.50		0.68		0.58
Oriental square			0.78		0.75		0.76
Sephardic cursive			0.99		0.70		0.82
Sephardic semi-square			0.71		0.88		0.79
Sephardic square			0.99		0.72		0.84
Yemenite square			1.00		0.46		0.63
**Accuracy**							0.68
**Macro avg**			0.73		0.70		0.69
**Weighted avg**			0.74		0.68		0.68

**Table 8:** Evaluation results of the hierarchical model at page level with 9 patches sampled per page.

Label			Precision		Recall		F1-Score
Ashkenazi cursive			1.00		0.89		0.94
Ashkenazi semi-square			0.92		1.00		0.96
Ashkenazi square			1.00		1.00		1.00
Byzantine semi-square			0.58		0.92		0.71
Byzantine square			0.52		0.79		0.63
Italian cursive			0.90		0.90		0.90
Italian semi-square			0.33		0.33		0.33
Italian square			0.50		0.20		0.29
Oriental semi-square			0.50		0.92		0.73
Oriental square			0.75		0.75		0.75
Sephardic cursive			1.00		0.75		0.86
Sephardic semi-square			0.79		0.92		0.85
Sephardic square			1.00		0.78		0.88
Yemenite square			1.00		0.48		0.65
**Accuracy**							0.75
**Macro avg**			0.78		0.76		0.75
**Weighted avg**			0.78		0.75		0.74
